# HIV risk among young Ghanaians in high school: validation of a multidimensional attitude towards condom use scale

**DOI:** 10.1080/02673843.2014.963629

**Published:** 2014-09-29

**Authors:** Rainier D. Masa, Gina A. Chowa

**Affiliations:** ^a^ School of Social Work, University of North Carolina, Chapel Hill, NC, USA; ^b^ Centre for Social Development in Africa, University of Johannesburg, Johannesburg, South Africa

**Keywords:** instrument validation, condom use, health belief model, confirmatory factor analysis, junior high school, Ghana

## Abstract

Condom use remains low among sexually active youth in sub-Saharan Africa. Theoretical and empirical evidence suggests that attitudes towards condom use are important predictors of actual condom use. However, few attempts have been made to systematically develop a valid scale that measures attitudes towards condom use among youth, particularly high school students in sub-Saharan Africa. Using the health belief model, we developed an instrument that measures such attitudes. We analysed survey data collected from 6252 Ghanaian junior high school students. We assessed construct validity using confirmatory factor analysis. Results indicate that attitudes towards condom use among young Ghanaians are best represented by a multidimensional construct. Young Ghanaians differentiate constructs related to perception of benefits and barriers to condom use, as well as perception of severity and susceptibility to HIV. This instrument offers a valid tool for assessing high school students' attitudes towards condom use and their HIV risk.

## Introduction

Young people in sub-Saharan Africa (SSA) face various sexual health risks as they transition from adolescence to adulthood. For instance, in Ghana a combination of being sexually active in adolescence and low condom use among sexually active youth heightens the risk of acquiring HIV and other sexually transmitted infections (STIs; Ghana Statistical Service [GSS], Ghana Health Service, & ICF Macro, 2009). In 2011, an estimated 1.9% of Ghanaian youth, aged 15–19 years, were living with HIV, an increase of 1.1% from 2010 (Ghana AIDS Commission, 2012). Young Ghanaians, aged 15–19, have the second highest prevalence of STI in the country, with 22% reported having STIs or STI symptoms (GSS et al., 2009). Despite high knowledge of condoms and HIV/AIDS, condom use among sexually active young Ghanaians remains low (GSS et al., 2009; Sallar, 2008–2009). Among sexually active youth, aged 15–19, 28% reported using condoms the first time they had sex and 30% reported using condoms during their last sexual intercourse (GSS et al., 2009). Low condom use among young Ghanaians is consistent with a pattern of low condom use among young people in SSA (Delva et al., 2010; Mthembu & Ndateba, 2012). The low condom usage, despite high knowledge level of condoms and HIV, suggests that other factors may influence young people's decisions to use or not to use a condom during sexual intercourse.

Although numerous factors influence sexual behaviours, theoretical and empirical evidence suggests that attitudes are highly related to sexual behaviours, including condom use (Albarracín, Johnson, Fishbein, & Muellerleile, 2001; Sheeran, Abraham, & Orbell, 1999). This pattern of evidence holds true among youth in Ghana and elsewhere in SSA, as research suggests that attitudes towards condoms predict condom use (Adih & Alexander, 1999; Kabiru & Orpinas, 2009). Given that attitudes may contribute to the low rate of condom use in SSA, an examination of young people's attitudes towards condoms may inform development of more relevant programmes to encourage young people to consistently use condoms and, ultimately, to prevent new cases of HIV.

Despite the widespread attention devoted to condom use among young people in SSA, few attempts have been made to systematically develop a valid and reliable scale that measures young people's attitudes towards condom use. To date, there are a few factor-analytically derived, multidimensional condom attitude scales validated with youth populations in SSA. Furthermore, fewer scales have been developed and validated exclusively with high school students, although many young people engage in their first sexual intercourse during high school and an increasing number of high school students are becoming sexually active. For instance, in Ghana, the age when many young Ghanaians engage in their first sexual intercourse corresponds with the time when they are in high school or of high school age (Doku, 2012). Based on recent available data, 6% of youth had their first sexual intercourse before the age of 15, while 37% had their first sexual intercourse before the age of 18 (GSS et al., 2009).

The main objective of this study is to develop and validate a theory-driven scale that measures attitudes towards condom use among high school students in Ghana. Using a large sample of Ghanaian junior high school students, the study aims to confirm the factor structure and quality of the attitudes towards condom use scale by demonstrating that the scale items are adequate indicators of hypothesised latent variables, that these latent variables measure distinct dimensions and that the dimensions are substantively consistent with theory and prior research.

### Measurement of attitudes towards condom use

A number of studies have developed and tested scales that measure different dimensions of condom attitudes including self-efficacy (Asante & Doku, 2010), barriers to condom use (Sunmola, 2001), benefits of condom use (Hanna, 1999) and susceptibility to HIV and other STIs (Zometa et al., 2007). Some studies have also developed and validated multidimensional condom attitude scales that combine two or more of the dimensions stated above (e.g. Helweg-Larsen & Collins, 1994). Results of these prior studies suggest that young people's beliefs about condoms are best represented with a multidimensional construct. In other words, young people discriminate between different dimensions of condom use attitudes and each dimension may influence condom use in a consistent or contradictory manner.

However, fewer attempts have been made to develop and validate multidimensional condom attitude scales with high school students in SSA, a population that is sexually active with low rates of condom use. Prior studies in SSA have focused mostly on convenient samples of college students (e.g. Asante & Doku, 2010; Madu & Peltzer, 2003) or a specific dimension of condom attitudes such as barrier (Sunmola, 2001) and self-efficacy (Asante & Doku, 2010). In addition, prior validation studies in SSA have relied mostly on exploratory factor analysis (EFA). Few studies have conducted confirmatory factor analysis (CFA), which can perform a more rigorous and strict test of construct validity (Kaplan, 2009). To address these gaps in research, we developed the *attitudes toward condom use scale* (ATCUS), validated the new instrument with a large sample of Ghanaian junior high school students and used CFA to confirm the factor structure and quality of ATCUS. In the development of ATCUS, we consulted scales that were originally developed in the USA and other parts of SSA (e.g. Boileau, Rashed, Sylla, & Zunzunegui, 2008) and prior studies on condom use among youth in Ghana and other parts of SSA (e.g. Adih & Alexander, 1999; Volk & Koopman, 2001).

The development of ATCUS also addressed a common limitation in the instrument development process, that is lack of explicit theoretical framework. Failure to use theory in developing a scale may provide a limited description of the phenomenon being studied, which may result in an incomplete understanding of the relationships that exist among hypothetical constructs (DeVellis, 2012). We are aware of only two studies in SSA (Boileau et al., 2008; Stanton et al., 1999) that explicitly used a theory in developing an instrument to measure condom attitudes among youth. In our study, the health belief model (HBM) was used to guide item generation and formulation of proposed dimensions of ATCUS. HBM was selected because: (1) it remains one of the more commonly used theories of health behaviour in the area of adolescent sexual health; (2) it has been used in the development of previous condom attitude scales (e.g. Sacco, Levine, Reed, & Thompson, 1991); (3) it is multidimensional and stratifies individual beliefs into several dimensions, including perceived benefits, barriers, susceptibility, severity and self-efficacy (Champion & Skinner, 2008); and (4) evidence suggests applicability of HBM when examining correlates of condom use among young Ghanaians (Adih & Alexander, 1999). Our selection of HBM was also supported by prior research. Empirical evidence suggests that HBM dimensions, including perceived susceptibility (MacPhail & Campbell, 2001), barriers (Volk & Koopman, 2001), severity (Van Rossem & Meekers, 2011) and self-efficacy (Adih & Alexander, 1999), are associated with condom use among youth in SSA. In addition, we added perceived social support as another dimension of ATCUS. Perceived social support was included because prior research in SSA suggests importance of peer norms and adults' attitudes towards condoms on young people's beliefs (e.g. Harrison et al., 2012; Rijsdijk et al., 2012). Evidence also suggests that Ghanaian youth are more likely to use condoms if they believe that their peers and older adults in their lives approve condom use (Adih & Alexander, 1999; Adu-Mireku, 2003).

## Methods

### Scale development

A team of researchers, led by the authors, developed the instrument (ATCUS) following the guidelines outlined by DeVellis (2012). First, the HBM was selected as a theoretical framework to guide item development and measurement of construct of interest and, ultimately, to improve content validity of the scale. Second, we generated an initial item pool of 30 questions. Experts in youth sexual health behaviour and measurement reviewed the initial item pool and measurement format. The experts examined individual items for developmental appropriateness and for gender or language biases, as well as content validity. Third, we tested the revised item pool of 21 questions with a sample of Ghanaian junior high school students (*N* = 51) in Greater Accra. Cognitive testing (Willis, 2005) of the items and response options was also conducted with another 20 students. We analysed data from cognitive interviews to identify hard-to-understand questions and evaluated the validity of responses from the intended population. We also evaluated pilot test data to examine distribution of responses and individual item performance. Based on pilot test data, we revised the instrument. One item was deleted, and the final version of ATCUS used in this study included 20 items. We then collected data from a large sample of Ghanaian junior high school students as part of a youth development project to examine construct validity of ATCUS. ATCUS was administered by trained interviewers who are fluent in English and local languages. All research procedures were approved by the institutional review board at the University of North Carolina at Chapel Hill and the University of Ghana. All participants provided written consent. In addition to youth's written assent, parental consent was obtained from parents of participants under the age of 18.

Table 1 lists the proposed dimensions of ATCUS, as well as individual items in each hypothesised latent construct. Each item on the scale was assessed with a five-point Likert scale, commonly used in instruments that measure attitudes (DeVellis, 2012). The five options range from 1 (*disagree a lot*) to 5 (*agree a lot*).

**Table 1:** Proposed latent factors and observed indicators of the attitudes towards condom use scale.

Factor	Definition	Indicators/items
Perceived benefits	Youth's belief of the usefulness or value of condoms.	1. Condoms are effective in protecting against HIV/AIDS.
		2. Condoms are effective against sexually transmitted infections.
		3. Condoms are effective in preventing pregnancy.
Perceived barriers	Youth's belief of the obstacles that will stop them from using condoms.	4. Condoms reduce sexual pleasure.
		5. Condoms are unreliable because they can break.
		6. The price of condom is too high to use regularly.
		7. Condom use can be bad for health.
Perceived severity	Youth's belief of the seriousness of HIV/AIDS.	8. HIV/AIDS is deadly.
		9. HIV/AIDS cannot be cured.
		10. HIV/AIDS can prevent people from enjoying life.
Perceived susceptibility	Youth's belief of their chances of acquiring HIV/AIDS.	11. People my age can get infected with HIV/AIDS.
		12. People my age are worried that they might get HIV/AIDS.
		13. A person my age could get HIV/AIDS by having sex with someone without using a condom.
Perceived self-efficacy	Youth's belief in their ability to use condom.	14. Condoms are easy to use.
		15. Condoms are easy to buy.
		16. Suggesting using a condom with a partner when having sex is easy.
		17. Convincing a partner to accept using a condom when having sex is easy.
Perceived social support	Youth's belief on whether their peers and important adults in their lives approve condom use.	18. My friends think condoms should be used during sex.
		19. Adults in my life think condoms should be used during sex.
		20. My partner thinks condoms should be used during sex.

### Data and sample

For this study, we analysed baseline data (*N = *6252) from a large, countrywide youth financial inclusion and development project. This project investigates the potential of savings accounts as a tool for youth development and financial inclusion in Ghana and other developing countries. Baseline data were collected in May and June 2011. In this project, 100 schools were selected from 8 of Ghana's 10 regions. Within each school, 60–63 students were randomly selected to participate in the study. The study sample had slightly more girls (51%) than boys. Nearly 4 in 10 youth were in grade level 6. Three in 10 youth were in junior high school level 1 and 2. Mean age was 15.33 years.

### Data analysis

#### Confirmatory factor analysis

The analysis focused on the 20 items comprising ATCUS. CFA was used instead of EFA because the factor structure of ATCUS is already known and specified by the theoretical model and prior research that guided instrument development. Although CFA increases validity by replicating factor structure based on EFA results and improves psychometric properties (Noar, 2003), prior studies on condom attitudes scale among youth in SSA (e.g. Asante & Doku, 2010; Boileau et al., 2008; Sunmola, 2001) have relied primarily on EFA, which is insufficient to establish psychometric properties of an instrument. CFA was also used to test hypotheses regarding the nature of the dimensions of a latent variable (in this case attitude) and how scale items relate to dimensions by establishing whether the data support the proposed dimensions and how well each item measures the hypothesised dimensions. Mplus 6.1 was used to perform CFA because of Mplus' ability to handle characteristics of our data, including clustering of students in schools, missing data and ordinal-level variables (Muthén & Muthén, 2010). Mean and variance-adjusted weighted least squares (WLSMV) estimator was chosen as the estimation procedure in Mplus because data were ordinal (Bollen, 1989; Muthén & Muthén, 2010). The fit indices used to evaluate goodness of model fit included χ^2^, root mean square error of approximation (RMSEA), comparative fit index (CFI) and Tucker–Lewis index (TLI). For χ^2^, a value resulting in a non-significant *p*-value (*p* > 0.05) was considered a good fit (Bollen, 1989; Kaplan, 2009). However, because obtaining a non-significant χ^2^ value is difficult, given large sample sizes, we also used RMSEA, CFI and TLI to determine whether our data met established criteria for fit. Cut-off criteria for the other fit indices included (1) a value between 0.05 and 0.08 for RMSEA (Browne & Cudeck, 1993) and (2) a value ≥ 0.95 for CFI and TLI (Hu & Bentler, 1999).

#### Competing CFA models

Another advantage of CFA is testing of competing models to find the model of best fit. Instead of simply confirming a model through one test, CFA can test alternative models. Because multiple models may have adequate fit, demonstrating that one model not only fits the data well but also has superior fit compared to alternative models increases confidence in our findings. By testing various models, CFA can also provide additional information about a scale's dimensionality, which may have implications for appropriate use of a scale, alternative versions of a scale and further theorising in a particular area (Noar, 2003). The χ^2^ difference test was used to determine which of the competing (nested) models had better fit to the data. Because we used WLSMV as the estimation procedure, the χ^2^ difference testing was done using the DIFFTEST option in Mplus. Furthermore, we created a calibration sample to test competing, alternative models and to identify the best-fitting measurement model. Because we did not have access to data from a new or separate sample, half of the original sample was randomly selected to generate a calibration sample (*N* = 3119). After establishing the best-fitting model, we retested the final model with a validation sample to determine if the results based on a calibration sample would be replicated. The validation sample (*N* = 3121) included the other half of the original sample. However, 12 cases in the original sample were not included in the analysis because those cases had missing values on all items. Seven of the cases were included in the calibration sample and the other five cases were in the validation sample.

We specified four competing measurement models. All multidimensional models were tested with correlated latent constructs as suggested by prior research (e.g. Adih & Alexander, 1999; Cummings, Jetter, & Rosenstock, 1978). The four competing multidimensional models were:

1. Model 1 was a *four-factor model* based on HBM. Thirteen items were hypothesised to load on the four original constructs of HBM: perceived benefits, perceived barriers, perceived severity and perceived susceptibility (Cummings et al., 1978). As shown in Table 1, items 1–3 were hypothesised to load on perceived benefits, items 4–7 on perceived barriers, items 8–10 on perceived severity and items 11–13 on perceived susceptibility. Support for this model suggests that Ghanaian high school students discriminate between the four HBM constructs.
2. Model 2 was a *five-factor model* in which 17 items were hypothesised to load on five dimensions: perceived benefits, barriers, severity, susceptibility and self-efficacy. The five-factor model included the four main constructs of HBM and perceived self-efficacy construct, which was added later to HBM (Rosenstock, Strecher, & Becker, 1988). As shown in Table 1, items 14–17 were hypothesised to load on perceived self-efficacy. Support for this model suggests that Ghanaian high school students discriminate between the five HBM constructs. Retention of this model may also indicate that the four original constructs of HBM may not fully explain the phenomenon being studied. In other words, addition of self-efficacy may be necessary to explain condom use attitudes and behaviours in a sample of Ghanaian high school students.
3. Model 3 was a *six-factor model* in which 20 items were hypothesised to load on six dimensions: perceived benefits, barriers, severity, susceptibility, self-efficacy and social support. Perceived social support was included because prior research in SSA suggests the importance of peer norms and adults' attitudes towards condoms on young people's beliefs. As shown in Table 1, items 18–20 were hypothesised to load on perceived social support. Retention of this model suggests that Ghanaian high school students discriminate between the six hypothesised latent constructs. Support for this model may also indicate that the five HBM constructs may not fully explain the phenomenon being studied. Perceived social support may be an important factor in explaining condom use behaviours in Ghanaian high school students.
4. Model 4 was a *second-order factor model.* Consistent with the first model, 13 items were hypothesised to load on the four main constructs of HBM: perceived benefits, barriers, severity and susceptibility. However, this model suggested that all four factors were related to a higher order factor. In this model, the higher order factor was a latent variable that was not directly measured by the 13 observed indicators. Instead, the higher order factor influenced scores on the observed indicators indirectly through the four first-order latent constructs. In other words, the second-order model hypothesised that the four distinct but related constructs could be accounted for by one common underlying higher order construct (in this case perceptions about condoms). In comparison to the first model, this second-order factor model can provide a more parsimonious and interpretable model by putting a structure on the pattern of correlations between first-order factors (Rindskopf & Rose, 1988). Few attempts have been made to test a second-order factor model in validation studies of scales that measure condom attitudes among youth in SSA. Support for Models 1, 2 or 3 may indicate the possibility of a second-order factor model.

## Results

### CFA results

As presented in Table 2, all hypothesised models met at least one pre-established fit criteria. The model of best fit among the four multidimensional models was decided according to three criteria: theoretical consistency, parsimony and statistical evidence. All four models were consistent with theory and prior research. However, the four-factor and second-order factor models were more parsimonious contrasted with the five- and six-factor models. Furthermore, the five- and six-factor models only met one fit criteria (RMSEA; see Table 2) contrasted with the four-factor and second-order factor models, which met three fit criteria (RMSEA, CFI and TLI; see Table 2). Thus, statistical evidence suggests that the two latter models had better fit to our data. We retained Models 1 and 4 because they were more parsimonious and strongly supported by statistical evidence. The next procedure was to determine whether Model 1 or Model 4 should be retained. Because Models 1 and 4 were nested, we conducted χ^2^ difference testing to provide statistical evidence on which model fit our data better.

**Table 2:** Confirmatory factor analysis results of the attitudes towards condom use scale.

			Fit indices			
	*N*	df	χ^2^	RMSEA (90% CI)	CFI	TLI
Proposed models using calibration sample						
Model 1: four-factor model	3119	59	365.91	0.04 (0.04–0.05)	0.96	0.95
Model 2: five-factor model	3119	109	694.09	0.04 (0.03–0.04)	0.94	0.93
Model 3: six-factor model	3119	155	757.20	0.03 (0.03–0.04)	0.94	0.93
Model 4: second-order factor model	3119	61	388.35	0.04 (0.04–0.05)	0.96	0.95
Final model using validation sample						
Four-factor model	3121	59	324.58	0.04 (0.03–0.04)	0.96	0.95

#### Nested models

Result of the χ^2^ test for difference testing showed a value of 19.83 and two degrees of freedom. This change in χ^2^, given the corresponding change in degrees of freedom, was statistically significant (*p* <  0.001). Because the χ^2^ test for difference testing had a significant *p*-value, the model fit became statistically significantly worse. In this case, we retained the less parsimonious and restrictive Model 1 because it had better fit. Although Model 4 offers advantages as a second-order factor model, the statistically better fit of Model 1 outweighs the improvement in parsimony of Model 4. Thus, we retained Model 1 as our final model. Given that the relationships hypothesised by Model 1 (four-factor) had the best fit to our data, we also tested a one-factor model in which 13 items were hypothesised to load on one factor instead of four distinct factors. This additional test was conducted to determine whether the final CFA model is best represented by a one-dimensional construct. The one-dimensional model did not have good fit to our data (χ^2^(65) = 1062.72, *p < * 0.001, RMSEA = 0.07, CFI = 0.88, TLI = 0.85). This finding suggests that Ghanaian high school students differentiate among the four HBM constructs and ATCUS is not represented by a one-dimensional construct.

#### Four-factor model

**Figure 1:**
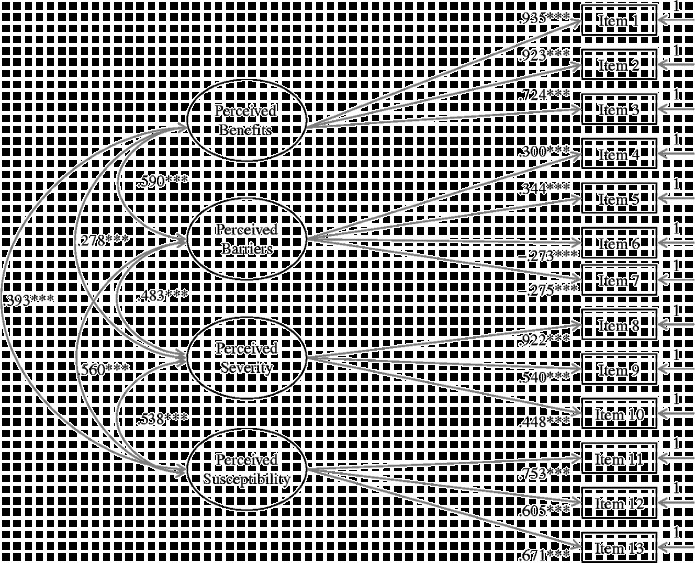
Four-factor model with calibration sample (*N* = 3119). ^*^^*^^*^ *p* <  0.001. Note: All model estimates are standardised.

**Figure 2:**
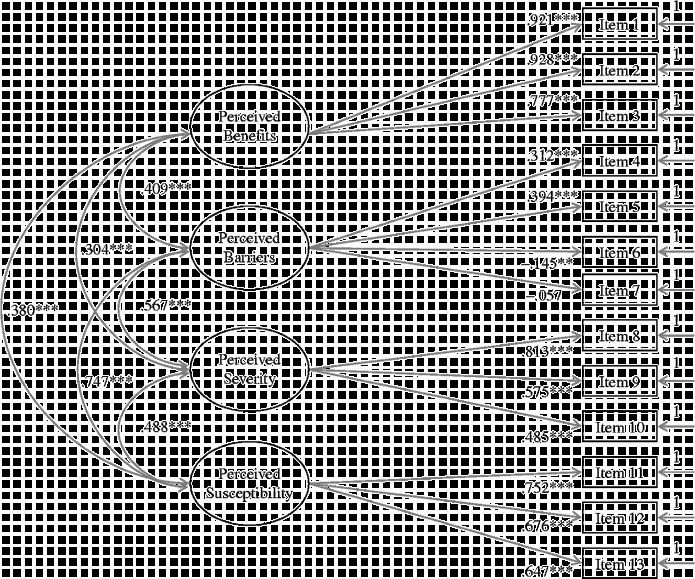
Four-factor model with validation sample (*N* = 3121). ^*^^*^ *p* <  0.01, ^*^^*^^*^ *p* <  0.001. Note: All model estimates are standardised.

We confirmed our final model with a validation sample. As presented in Table 2, results showed that model fit of the four-factor model with a validation sample was consistent with model fit of the four-factor model using a calibration sample. With the exception of the χ^2^ values, the values of the other fit indices for both calibration and validation samples were identical. The consistent pattern suggests that results of the model development process were replicated with an additional sample. Figure 1 illustrates the four-factor model with standardised factor loadings and inter-factor correlations based on a calibration sample. All factor loadings, variances and covariances were statistically significant (*p* <  0.001). All factor loadings were higher than the absolute value of 0.25. The estimates of factor variances were 0.88 for perceived benefits, 0.10 for perceived barriers, 0.85 for perceived severity and 0.57 for perceived susceptibility. Furthermore, inter-factor correlation coefficients ranged from 0.28 to 0.60. The *R* ^2^ values of individual items ranged between 0.07 and 0.88. Although there is no generally agreed upon cut-off for what is an unacceptable *R* ^2^, higher values are preferred because higher values signify that more of an indicator's variance is associated with the latent variable it is hypothesised to help measure. Three items had an *R* ^2^ greater than 0.80: items 1, 2 and 8. On the other hand, items that loaded on perceived barriers had the lowest *R* ^2^ (between 0.07 and 0.12). The remaining items had moderate *R* ^2^ values ranging from 0.20 (item 10) to 0.57 (item 11). Finally, the parameters (factor loadings, variances and inter-factor correlations of observed indicators) that were obtained using a calibration sample were consistent with the parameter values obtained using a validation sample. Figure 2 illustrates the four-factor model with standardised factor loadings and inter-factor correlations based on a validation sample. As presented in Figure 2, all but one critical feature of the model remained statistically significant. The factor loading of item 7 (‘Condom use can be bad for health’) was not significant in the validation sample. In summary, adequate fit of the final model was replicated and most parameters remained stable. Thus, statistical evidence suggested that the final (four-factor) model has been validated.

After determining the best-fitting model at the student level, we performed a multi-level CFA to account for the clustered nature of our data (i.e. students were nested within schools). Intraclass correlations (ICCs) for each observed item ranged from 0.05 to 0.16, with 9 of 13 items having an ICC of < 0.10. When ICC findings are close to zero or less than 0.10, a multi-level model may not be justified because a single-level CFA model will yield reasonable estimates (Julian, 2001; Muthén, 1997). Given these findings, we kept the single-level model as the final model.

## Discussion

Consistent with prior studies (e.g. Boileau et al., 2008; Helweg-Larsen & Collins, 1994), results suggested that attitudes towards condom use among young Ghanaians is best represented with a multidimensional construct. In particular, results provided clear support for the four-factor model. Retention of the four-factor model indicated that Ghanaian high school students differentiate between the four main HBM constructs and the phenomenon being studied may be fully explained by perceived benefits, barriers, severity and susceptibility. The multidimensionality of condom attitudes among young Ghanaians suggested that when explaining or predicting condom attitudes or behaviour, researchers and practitioners must specify which specific components of condom attitude will be considered.

Consistent with the HBM, our results suggested that perception of benefits and barriers of condom use and also perception of severity and susceptibility to HIV are important components of Ghanaian high school students' attitudes towards condom use. Results also indicated that the proposed latent factors measure distinct dimensions and that the dimensions are substantively consistent with prior research (e.g. Adih & Alexander, 1999; Volk & Koopman, 2001). In addition, variances of the four latent factors were significantly different from 0. Latent factors with significant variances are useful measures because they capture meaningful differences among Ghanaian high school students in our study. As expected from theory and prior research, the four main HBM constructs were significantly correlated. Youth who hold a positive attitude regarding the benefits of condom use may hold similar attitudes in other areas of condom use.

Results also suggested that most ATCUS items are adequate indicators of the proposed latent constructs. The magnitude of factor loadings was consistent with prior studies conducted among young people in SSA (e.g. Boileau et al., 2008). All but one factor loading based on different samples were statistically significant. Statistical evidence suggests that item 7 can be deleted. However, deletion of item 7 should be done with caution. There are varying opinions about whether model components should be removed due to non-significance. Some researchers believe that theoretically justified elements should remain in the model. Results also show that HBM constructs can be accurately assessed with fewer items. We used three to four observed indicators to measure the hypothesised dimensions. A smaller number of items in the scale might have reduced response burden on participants and allowed additional space to (1) assess other critical components of young people's attitudes towards condom use (e.g. perceived social support) and (2) more accurately represent the construct being studied.

Although study findings support a multidimensional ATCUS, empirical results do not support inclusion of two other latent factors: perceived self-efficacy and social support. Our data did not support a model with perceived self-efficacy and social support, perhaps because of the characteristics of our sample (e.g. age and lack of sexual experience), which may affect how young people develop their beliefs. In ATCUS, the items that measure perceived benefits, barriers, severity and susceptibility tap into knowledge about HIV/AIDS and condoms. Knowledge about HIV and condoms is so widespread or common among Ghanaian high school students that such knowledge becomes a norm. On the other hand, the other two factors measure knowledge or belief that may not be relevant, at least to our sample of Ghanaian high school students, because of their age, lack of sexual experience or simply because they do not know yet the answer to the items being asked. In other words, perceived self-efficacy and perceived social support may measure individual attitudes based on sexual experience contrasted with the four main HBM constructs, which may measure normative attitudes. Indicators aimed at measuring attitudes based on experience may not be relevant to sexually inexperienced high school students. Even though the current study did not support a multidimensional model with perceived self-efficacy and social support, our results did not indicate that these two factors are not predictive of condom use among youth.

The use of an explicit theory made the scale development process easier as theory offered specific suggestions on how to measure the construct of interest, including the proposed multiple dimensions. Consistent with prior studies (Boileau et al., 2008; Stanton et al., 1999), our results suggested that instruments developed around social cognition models may be useful and relevant for use in different cultural and geographical settings. In particular, results suggested that HBM, originally developed in the USA, may be as useful in African contexts as in western contexts for examining potential beliefs that shape young people's sexual behaviours, including condom use.

ATCUS can be useful in monitoring and assessing HIV risk among sexually active high school students or youth of high school age. The likelihood of using a condom by sexually active youth can be assessed using the four dimensions of ATCUS. With the exception of perceived barriers, a high score indicates more positive attitudes towards condom use, which in turn may predict higher likelihood of condom use. Higher levels of perceived benefits, severity and susceptibility are hypothesised to be associated with more condom use, while higher level of perceived barriers is hypothesised to be associated with less condom use. These four dimensions may also allow researchers and practitioners to specify which aspect of condom beliefs is most predictive of young people's intention and actual use of condom. Such prediction may lead to more effective messaging of condom promotion and other prevention interventions targeted to high school students in Ghana and elsewhere in SSA. Furthermore, CFA results suggested the appropriate method of using ATCUS for assessment. Practitioners often sum, average or otherwise combine the scores of a set of assessment items and use the new score for decision-making. However, such practice may not be appropriate with ATCUS. Rejection of the second-order factor model suggested that the factors, although correlated with each other, are not related to a higher order factor. Weak statistical evidence existed to support summing the total of the entire scale, and if such an option were used, results might not represent a meaningful score. Practitioners may find it more useful to examine the subscales individually than to meaningfully interpret the total ATCUS score.

Knowledge of condoms alone may not be adequate to change attitudes and to encourage sexually active youth to use condoms. Condom education programmes that include normative and experience-based attitudes may be helpful in increasing condom use among sexually active high school students in Ghana. Health education messages for Ghanaian youth must combine threats of susceptibility and severity of HIV with information about effectiveness of condoms in preventing HIV infection. Prevention programmes should also address ways to overcome barriers to condom use, such as inaccurate knowledge and harmful beliefs. Addressing underlying attitudes towards condom use may also illustrate a more holistic picture of the problem and provide effective ‘entry points’ for programme development and policy intervention. Although condom education may be controversial in certain communities because some may argue that it promotes early sexual experimentation, majority of Ghanaians support condom use education for youth, including those aged 12–14 years (GSS et al., 2009). Stakeholders should capitalise on this support to develop and implement programmes that addresses young people's condom use attitudes, particularly those that may contribute to adverse outcomes.

The study has limitations. First, HBM may not account for other factors that may explain condom use among youth (Helweg-Larsen & Collins, 1994; Lollis, Johnson, & Antoni, 1997). In addition, social cognition models such as HBM have been criticised for being overly focused on individual decision-making. Theoretical models that rely on rational choice behaviours ignore broader social, economic and cultural factors that operate outside an individual's ability to weigh costs and benefits but have substantial influence on sexual behaviours of young people in SSA. Second, results do not tell us anything about the validity of HBM. Similarly, results do not provide guidance on which of the proposed dimensions are more predictive in shaping positive behaviour. Third, because of data limitations, we were not able to test criterion-related validity. Fourth, our results do not provide evidence on whether ATCUS performs the same across two or more populations. For instance, we do not know whether the psychometric qualities of ATCUS are the same for young men and women. Similarly, our sample only included in-school youth, and so, we do not know ATCUS' validity when used with a sample of out-of-school youth.

## Conclusions

The importance of attitudes in explaining and predicting condom use suggests that reliable and valid measures exclusively developed for young people are critical to understand underlying factors that influence condom use. Valid and reliable scales are also important for improving research findings and guiding development of interventions targeted to young people, a population seen as having high risk to HIV but also viewed as a population in which prevention interventions may be most effective. In this study, we presented the model development process of an instrument designed to measure attitudes towards condom use among Ghanaian high school students and tested the construct validity of its components. This study also addressed limitations of prior studies, such as limited development and validation of condom use scales for high school students in SSA, non-use of theory in guiding scale development and non-use of CFA in establishing construct validity. In conclusion, ATCUS is a valid instrument to investigate the role of sociocognitive factors in condom use among youth. ATCUS may be helpful in identifying subgroups of youth at greater risk of contracting HIV and determining the effectiveness of efforts to promote positive attitudes towards condom use and to increase condom use among sexually active youth.
